# Particulate Matter Contributions from Agricultural Tilling Operations in an Irrigated Desert Region

**DOI:** 10.1371/journal.pone.0138577

**Published:** 2015-09-30

**Authors:** Meilan Qi, Kairong Lin, Xiangzhen Li, Ted W. Sammis, David R. Miller, Junming Wang

**Affiliations:** 1 School of Science, Wuhan University of Technology, Wuhan, Hubei, P. R. China; 2 Department of Water Resources and Environment, Sun Yat-sen University, Guangzhou, Guangdong, China; 3 Key Laboratory of Mountain Ecological Restoration and Bioresource Utilization, CAS, Ecological Restoration Biodiversity Conservation Key Laboratory of Sichuan Province, Chengdu Institute of Biology, Chinese Academy of Sciences, Sichuan, China; 4 Department of Plant and Environmental Sciences, New Mexico State University, Las Cruces, New Mexico, United States of America; 5 Department of Natural Resources and Environment, University of Connecticut, Storrs, Connecticut, United States of America; 6 Climate and Atmospheric Science Section, Illinois State Water Survey, Prairie Research Institute, University of Illinois at Urbana-Champaign, Champaign, Illinois, United States of America; Peking University, CHINA

## Abstract

Sources of regional particulate matter (PM), particularly agricultural operations, must be understood in order to manage the air quality in irrigated dry climates. Direct monitoring measurements alone are useful, but not sufficient, to estimate regional PM source concentrations. This paper combines modeling with ground (point) and airplane (spatial) measurement methods to estimate regional PM10 (PM diameter≤10 μm) contributions from agricultural operations. Hourly data from three air quality monitoring stations positioned at a 2-m height located on the west and east mesas of New Mexico’s Mesilla Valley and in the valley at Anthony, NM were acquired from the New Mexico Air Quality Bureau. The study spanned the agricultural tilling season, March 1 to April 30, for the years 2008 to 2012. One- second spatial PM10 concentrations at 200 m above the valley floor were measured during a two-hour controlled field tilling operation on April 1, 2008. The HYSPLIT 4.0 (Hybrid Single-Particle Lagrangian Integrated Trajectory version 4) model was run at the corresponding times and heights, outputting PM10 concentrations from all potential agricultural tilling operations. The calculated percentage contribution (modeled PM10 concentration/measured PM10 concentration) indicated that the near-surface (2-m height) proportion from the agricultural operations for five seasonal averages ranged from 0.7% to 1.5% on the west and east mesas and 1.3% for the valley site at Anthony. There were 71 hourly high values of contribution ratios ranging from 30 to 100% at the three sites, depending on the wind speed and direction.

## Introduction

### Background

Particulate matter of an aerodynamic diameter less than or equal to 10 microns, PM10, is regulated by the U.S. Environmental Protection Agency (EPA) as part of the National Ambient Air Quality Standards. Different sources can contribute to the regional PM10, such as factory stacks, agricultural operations, construction sites, unpaved roads, wood/waste burning, or concentrated animal feeding operations. For air quality management purposes, it is important to know which percentage of each source contributes to the regional PM10 concentrations. The EPA declared Anthony, in Dona Ana County, NM, a nonattainment area for PM10 levels in 1991 [1]. Dona Ana and Luna counties in New Mexico each developed a Natural Events Action Plan (NEAP) for PM10 exceedances caused by uncontrollable natural events. Both plans specify that adequate dust control management plans must be executed [2]. Local ordinances were implemented to control dust from construction sites, but dust management from agricultural operations is lacking because no studies have demonstrated the contribution of agricultural practices to regional PM10 levels. In addition, no plans were made to pave the numerous unpaved county roads, which also contribute to regional PM10 levels. Consequently, the NEAP plans include the development of educational efforts and dust warning systems, but they contain only limited dust-control measures and only for construction sites. Measurements of dust from agricultural sources, construction sites, and unpaved roads are difficult to determine because air quality monitoring measures only the total concentration. In many cases in the western United States, most dust sources cannot be controlled; yet EPA requires that when its standards are exceeded, those sources that can be reduced are regulated. However, regulations for unpaved roads and agricultural dust have been excluded because of a lack of studies showing the contribution of these sources to overall regional dust levels.

**Fig 1:**
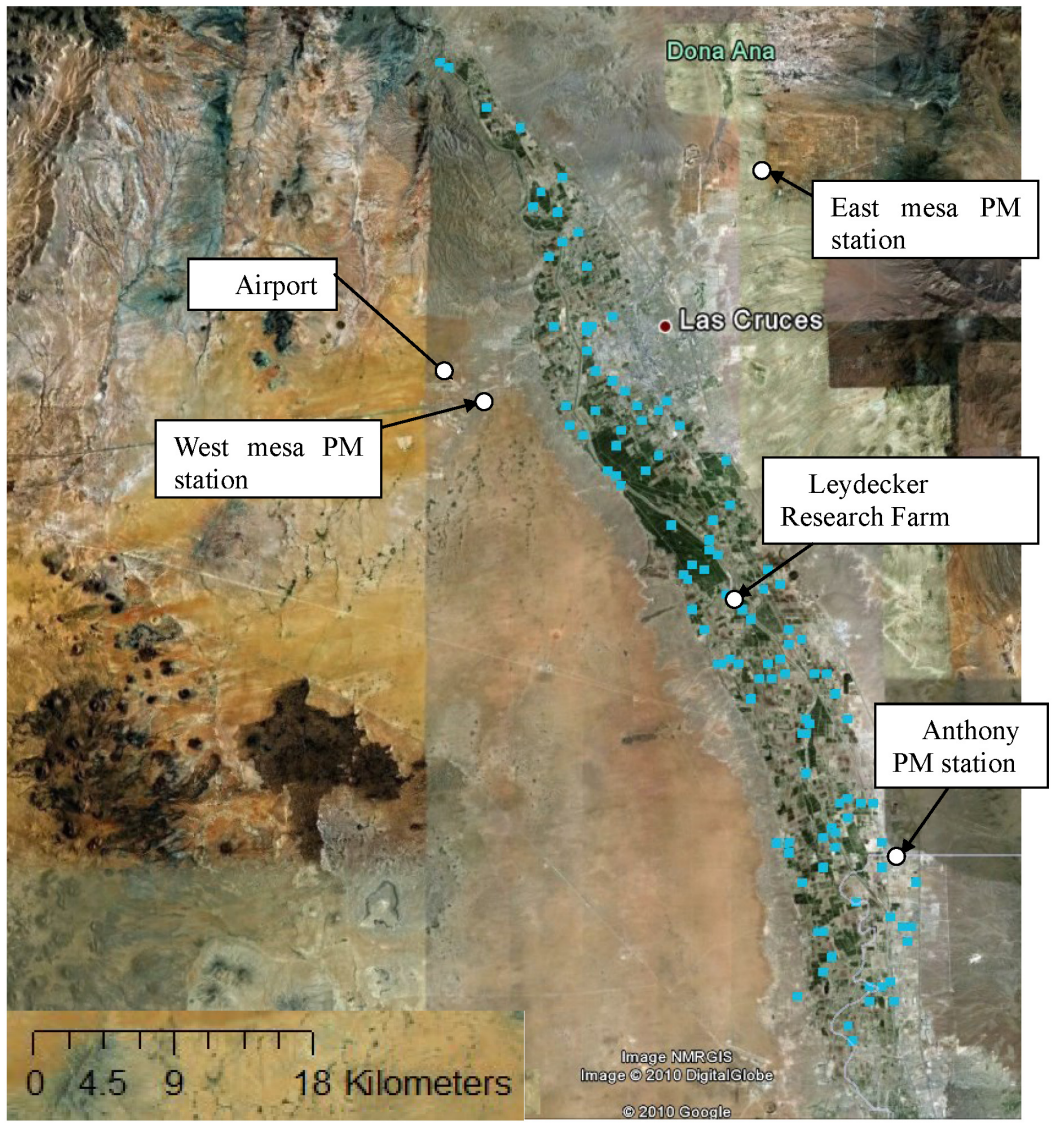
The potential tilling sources (blue squares) in the Mesilla Valley of southern New Mexico.

The state of New Mexico monitors the PM10, and in some cases the PM2.5 levels, at air quality sites throughout the state. In the Las Cruces, NM area, one site is on the east mesa where a majority of new housing development is occurring, and a second site is on the west mesa where housing development occurs at a lower, but continuously expanding, rate (Fig 1). The state also monitors PM10 concentrations in Anthony, south of Las Cruces in the Mesilla Valley, and at several sites near the New Mexico/Texas border. People living in the valley have dealt with dust from agricultural operations since 1918 when extensive irrigated agriculture arrived. However, homeowners on the east and west mesas are now becoming concerned about the PM10 concentrations’ impact on their health.

Recently, PM2.5 concentrations in the air have been determined to be a critical health risk, and sensors are gradually being converted from PM10 to PM2.5. However, in New Mexico and other western states, most measurement sites are still measuring only PM10 concentrations in the air.

### Air Quality Models

A number of air quality models have been developed for regulatory, policy, and environmental decision-making and scientific studies. The most common air dispersion models are steady-state, straight-line, Gaussian plume models, such as the ISCST3, which calculates contaminant concentrations for each hour, assuming meteorological conditions are uniform across the modeling domain [3]. More complex models, such as the CMAQ [4], represent a comprehensive treatment of meteorology, emissions, and chemistry. These models require considerable expertise and computer resources to set up, run, and interpret the results. Air quality models that are more complex than the simple Gaussian plume models, but less complex than the CMAQ, include the CALPUFF [5], the HYSPLIT 4.0 [6–7], and the AERMOD [3] models. Each model has advantages and liabilities. Puff models (e.g., CALPUFF) use a Lagrangian formulation of advection to distribute discrete puffs of emissions that expand in size and shape according to a Gaussian plume model. HYSPLIT 4.0 can be run as a puff model or as a particle model in which a fixed number of particles are advected in the model domain by the mean wind field and spread by a turbulent component. AERMOD is an example of a complex Gaussian model, which is an improvement on the ISCST3 model in which concentration distribution in a stable boundary layer is modeled by a Gaussian process in both vertical and horizontal directions. The concentration distribution in the convective boundary layer assumes a Gaussian horizontal distribution, but the vertical distribution is described by a bi-Gaussian function. All of these models, except the HYSPLIT 4.0, have a longer time step than desired, usually one hour, because wind vector data are not available on a finer time step. Also, the horizontal distribution of meteorological data with vertical profiles is usually gleaned from simulated climate data with a 12-km grid spacing. Finer horizontal grid climate data (2.5 km) without vertical profiles are available on the Internet, but the interpolated data at the finer grid have not been verified. The interpolated data cannot be used for most of the mentioned models because the data do not include vertical profiles.

The HYSPLIT 4.0 is easy to use and has been verified to simulate PM10 dispersion from agricultural operations in the study areas used in this paper [6,8–9]. The HYSPLIT 4.0 model was developed by the National Oceanic and Atmospheric Administration (NOAA) Air Resources Laboratory [7]. This dynamic model can have a simulation time step of one minute to one hour or more. The HYSPLIT 4.0 can ingest NAM12km climate forecast data from the North American Mesoscale (NAM) model, which produces NAM12km regional data with a vertical profile from 0- to 1,000-m heights with 12 km of horizontal resolution and a three-hour frequency. The data available at and above 50 m include wind speed and direction, air temperature, relative humidity, and total kinetic energy. The surface layer data include mean sea-level pressure, three-hour accumulated precipitation, three-hour accumulated convective precipitation, 2-m temperature, 2-m relative humidity, 10-m wind speed, surface pressure, latent heat net flux, sensible heat net flux, friction velocity, surface roughness, downward shortwave radiation flux, and surface height. Based on these data, the HYSPLIT 4.0 calculates turbulence using the Kanthar/Clayson method [7] in its simulations.

All air quality models require source point strength emission data. Quite a few studies provided PM10 emission factors and simulation models for agricultural tilling operations. For example, [10–11] measured PM10 emission factors for tilling operations in California’s San Joaquin Valley using point samplers and Light Detection and Ranging (LIDAR) equipment. [12] measured and provided a model that can calculate emission factors from the independent variables of crop type, tilling type, soil moisture, and silt content. Local field-scale PM10 dispersion models (e.g., [13]) were developed to assess the risks of PM10 dispersal that tractor drivers and people living in nearby houses experience.

Studies on tilling operation emissions and dispersions exist, but there is a lack of studies that provide the percentage of contributions from agricultural tilling operations to the total regional PM10. The objective of this study was to combine air quality monitoring station data and the HYSPLIT 4.0 model to evaluate the percentage of contributions from agricultural tilling operations in the Mesilla Valley region of southern New Mexico to the regional PM10.

## Materials and Methods

Total PM10 data were measured hourly at three ground sites (2-m height) in March and April in 2008–2012, and spatial total PM10 data were measured by an airplane (at 200-m height) during a two-hour period on April 1, 2008, in Las Cruces. Next, the HYSPLIT 4.0 model calculated PM10 concentrations at 2 m and 200 m from all potential agricultural operations during the corresponding periods. Finally, the calculated concentration was compared with the corresponding ground 2-m height measured data and airplane measured data at a 200-m height, and the contribution ratio (calculated/measured) was obtained.

### HYSPLIT 4.0 Model Description

The HYSPLIT 4.0 for particle sources is based on a Lagrangian model system. The advection of particles is computed from the average of the three-dimensional velocity vectors for the initial position P(t) at time t, and the first-guess position, P'(t + Δt), at time t + Δt. The velocity vectors V(P,t) are linearly interpolated in space and time. The first guess position is

P'(t+Δt)=P(t)+V(P,t)Δt, (1)

and the final position is

P(t+Δt)=P(t)+0.5[V(P,t)+V(P',t+Δt)]Δt. (2)

In addition to the advective motion of each particle, a random component to the motion is added at each time step (t) according to the atmospheric turbulence at that time. In this way, a cluster of particles released at the same point will expand in space and time, simulating the dispersive nature of the atmosphere [14]. The model also includes deposition algorithms based on wind speed and particulate settling speed. Details of the algorithms are described in [7].

#### Model inputs

NAM12km data from March 1 through April 30 each year 2008 to 2012 were used for the meteorological data inputs to compare with the measured 2-m air quality point data from the New Mexico Air Quality Bureau. In addition, a simulation run was conducted on April 1, 2008, using NAM12km data to compare with the corresponding measured spatial 200-m height data collected from an airplane, as described below. The source strength (mg/hour) must be known in order to output the absolute concentration. The source strength data (2,028,000 mg/hour) were measured before the experimental day and were published in [12]. They were measured in Las Cruces on March 13, 2008 and represent the source strength from disking operations under dry soil moisture conditions (Soil moisture: 0.0234 g H2O/g soil; silt content: 0.57 g silt/g soil). Because there was no rain from March 13, 2008 to April 1, 2008 (this experiment), the soil moisture was still very dry on April 1 (similar to and possibly dryer than the March 13 soil moisture). The source strength was the largest for plowing, disking, leveling, listing, and planting operations obtained in Las Cruces in 2005 and 2008 experiments, as described in [8,12]. Therefore, the contribution simulations in this study represent the maximum contributions from field preparation in agricultural operations.

#### Model parameters

The parameters of the HYSPLIT 4.0 for PM10 simulation at Las Cruces were optimized and verified in [8]. The parameters were as follows: The settling speed of the dust particles from the disking operation was set to 0.000038 to 0.006 m/s (Appendix 1), based on the seven particle sizes. The seven bin sizes of the particles used by the HYSPLIT 4.0 in this study are specified in the input file (Appendix 1) and were measured in an agricultural operation dust experiment near Las Cruces where disking operations were conducted during dry soil conditions (soil moisture: 0.28 g H2O/g soil; silt content: 0.57 g silt /g soil) [15].

The released particle number in the model was set to 25,000. The output grid was set with a 0.003-degree spacing in longitude and latitude (i.e., 0.282 km east-west spacing and 0.333 km north-south spacing). The model ingests 12 km data for climate variables and then interpolates output results to the specified grid spacing. The finer the input climate grid, the more accurate the finer output grid becomes. However, as stated above, the 12-km grid input climate data interpolated to the finer 2.5-km grid has not been verified and does not include vertical meteorological profiles, and thus cannot be used in the simulation.

The height of the source data was set at 5 m because the disks were 1 m in height, and the centrifugal force from the disks throws the particles up to a height of 5 m before the dust cloud starts to move downwind as verified and optimized in [8]. Other model inputs are presented in Appendix 1.

#### Model output

The model outputs hourly-averaged spatial concentrations (mg/m^3^) at defined grids for the March-April season and one-minute concentrations for the airplane experimental period.

### Potential sources of agriculture tilling dust

To represent the regional agricultural operations at the time of the observations, an identical source was distributed randomly to each 138-ha agricultural area (Fig 1). The reasons for this source size are described in the next section.

### Total emission estimates

Conducting the simulations required estimating the number of tractors working at the same time in the Mesilla Valley. The valley has 40,130 acres (16,240 ha) of agricultural fields [16]. Every year in March and April, tilling operations are conducted, and dust problems are most serious because the weather is windy in the spring. It is assumed that each tractor works 84 hours each week, 12 hours each day, (for a total of 672 hours in two months) and can till 2.5 acres per hour (1 ha per hour) (the estimate is based on the personal communication with New Holland Tractors Dealership, El Paso, TX and surveys from farmers at Las Cruces). Completing five operations (plowing, disking, leveling, listing, and planting) requires 120 tractors (= 16,240×5/1/672). Each tractor works an area of 138 ha (16,240/120). Therefore, in the simulations, a random point source was set for the HYSPLIT 4.0 in each 138-ha agricultural area (Fig 1). Tractor locations shift daily, and it is not possible to measure the location and number of tractors operating on a specific day, so the approximation method described was used to calculate the number and location of source activity. This estimate may be erroneous, which would pose an error in the amount of dust contributed by agricultural operations, but the estimate is the best first approximation of the upper limit of agriculture field dust emissions until Global Positioning System (GPS) units are installed on all tractors and the exact location of tractor operations can be determined.

### Superimposition for ground sites

The HYSPLIT 4.0 model was run to simulate PM10 dispersion from a tilling source field at the Leyendecker Research Farm in Las Cruces (32° 11′ 50.19″ N, 106° 44′ 18.76″ W, elevation 1,173 m) for every hour of each day from March 1 to April 30 for the years 2008–2012, from 00:00 to 24:00 MDT (MDT: Mountain Daylight Time). In the model, the dust release for farming operations was from 08:00 to 20:00 MDT. The model output was obtained for a simulation altitude of 2 m, corresponding to the height of the New Mexico air quality sensors.

Once the potential source locations were identified for each source point, the single-source dust model that runs every hour at 2 meters was superimposed on the source locations and added to derive the concentrations for each output grid. The superimposed grid was 0.282 km east-west and 0.333 km north-south. Note that topography was not accounted for in the model, and the variability of the wind field was not completely represented. Thus discussion of small-scale plume dynamics from multiple sources was inhibited, but the regional focus of the study was not degraded.

Hourly PM10 data from three air quality monitoring stations at a 2-m height located on the west mesa (32° 16' 14.7" N, 106° 54' 18" W) and on the east mesa (32° 25' 4.584" N, 106° 41' 1.320" W), away from the valley, and the Anthony station in the valley (32° 0' 14.364" N, 106° 36' 20.977" W) were acquired from the New Mexico Air Quality Bureau (New Mexico Air Quality Bureau Air Quality Website, 2013c) from March 1 to April 30 each year in 2008 to 2012. The PM10 data were collected using a TEOM™ 1405-D Continuous Ambient Particulate Monitor (Thermo Scientific™, Waltham, MA). The calculated proportion of the contribution (modeled/measured PM10 concentration) at the three sites indicated the contribution that comes from the agricultural operation as a percentage of that from all sources. Wind rose data (hourly wind speed and direction) also were acquired from the three New Mexico air quality sites (New Mexico Air Quality Bureau Air Quality Website, 2013c) to understand why the contribution from agricultural operations varied from hour to hour and day to day.

### Airplane Experiment

The three mentioned PM10 sites measured point data at fixed locations. To evaluate the HYSPLIT model and supplement the data, 1-s spatial PM10 data at 200 to 550 m were measured using an airplane (with an average speed of 70 MPH) on April 1, 2008.

The experiments were conducted at the Leyendecker Plant Science Research Center at New Mexico State University in Las Cruces, New Mexico (32° 11′ 50.19″ N, 106° 44′ 18.76″ W). A tractor was continuously working in a cotton field (200 × 100 m), and disking was performed from 11:13 to 13:13 MDT (16:13 to 18:13 UTC).

**Fig 2:**
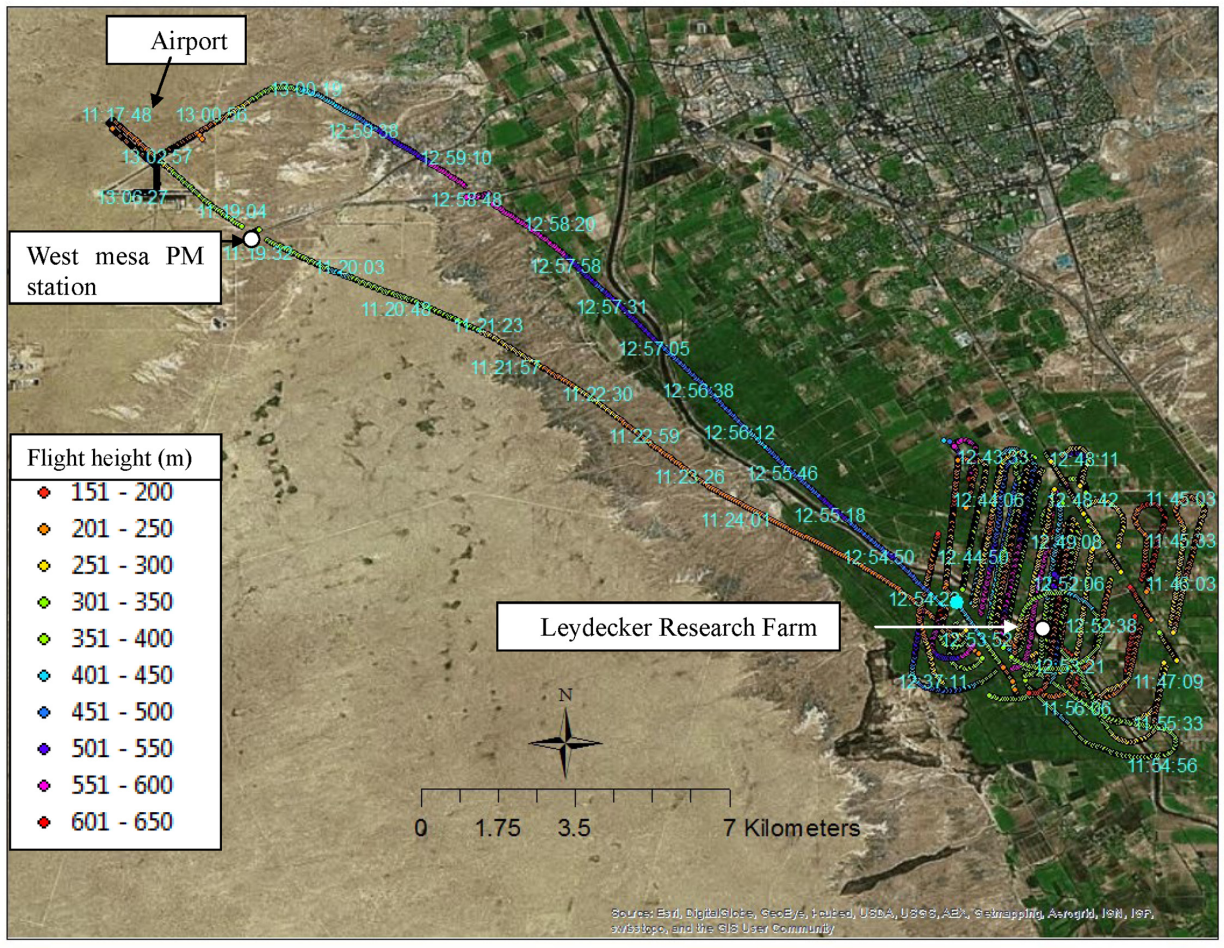
Airplane GPS tracks during the regional PM10 experiment on April 1, 2008, at Mesilla Valley, Las Cruces, NM (colored points: 1-s tracks). The labels are the time of the points.

Fig 2 shows the map of the experimental area and the airplane (a Cessna) tracks recorded by a GPS. The figure shows the airplane locations at 1-s intervals recorded by a GPS sensor (010-00321-00 GPS 18 Deluxe USB Sensor for Laptops, Garmin, Olathe, KS). A PC laptop was used to record 1-s position data, including date, time, altitude, and location as longitude and latitude. The horizontal and vertical accuracy of the GPS unit was *<*15 m.

PM10 data were measured by a DustTrak sampler (Model 8520, TSI, Inc., Shoreview, MN) mounted on the airplane. The DustTrak sampler had a flow rate of 1.7 L min^−1^ and made measurements at 1-s intervals. The sampler was factory calibrated, and the flow rate was calibrated (1.7 L min^−1^) prior to conducting the experiments. The flow rate of the DustTrak was kept at 1.7 L min^−1^ during the Dustrak sampling. Therefore, the airplane speed did not significantly affect the sampling. The Cessna airplane made multiple passes in the area shown between a 200-and 550-m elevation and sampled PM10 concentrations. Each pass was for a specific height (e.g., 200 m), and the height varied with time (Fig 2). Data in the height range (desired height±25 m) were used for the desired height (e.g., 200 m) data analysis. The average airplane speed was 70 miles h^−1^ and the speed was slower when it made turns. The wind speed and wind direction data were measured at 10 Hz, and the Dustrack sampler had a sampling frequency of 1 s. Based on the time stamps of the data, the data points were synchronized at 1-s intervals for data analysis. The source strength mainly depended on the parameters of soil moisture, silt content, tractor speed (fixed speed and did not change much during experiments), and tractor and tilling equipment type. Because these parameters did not change or did not change significantly, the source strength did not change much during the sampling period.

For evaluation, the HYSPLIT 4.0 model was run from 11:13 MDT (farming start time) to 13:13 MDT (the end of the airplane sampling time) using the NAM12km data on April 1, 2008, from the Leyendecker Research Farm. The model output was run for a simulation altitude of 200 to 550 m to compare the results to airplane measurements at that height. The concentration data at a 200- to 550-m height from 20 min before the disking operation was started were separated as the background data. These data were acquired while the plane traveled from the airport to the field location, a distance of 21 km, and then the plane circled the field area. The data average (0.0048 mg m^−3^) at 200 to 550 m before entering the experimental field area was used as the background; i.e., the other measured data removed the background value before comparing it with the model simulations. Details of the verification process were described in [8].

After the model’s evaluation, a single-source run at a 200-m output height from 08:00 to 11:13 MDT was superimposed on all potential tilling sources in the Mesilla Valley. The superimposed concentrations (from all tilling operations) were divided by the corresponding airplane measurements (contributed from all potential PM10 sources) to obtain the PM10 contribution ratio. Only the 200-m height data were used to calculate the PM10 concentration ratio because this was closest to ground level where human activities occur and contained the highest concentrations of dust compared to other airplane sampling heights.

## Results and Discussion

### The general characteristics of simulations

**Fig 3:**
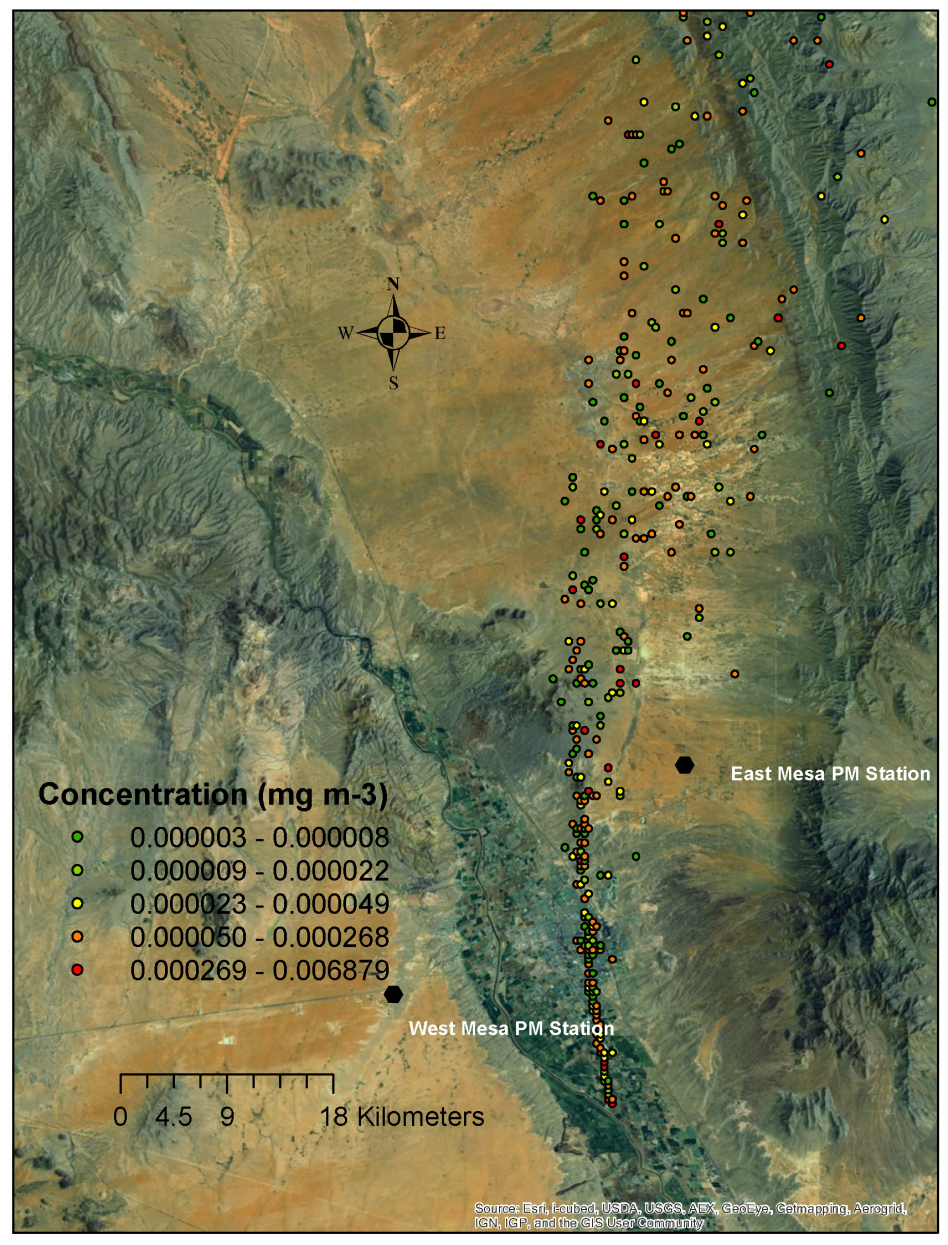
PM10 concentration plume from a single tilling source at 13:00 MDT on April 1, 2008, in the Mesilla Valley of southern New Mexico at a 2-m height (MDT: Mountain Daylight Time).

**Fig 4:**
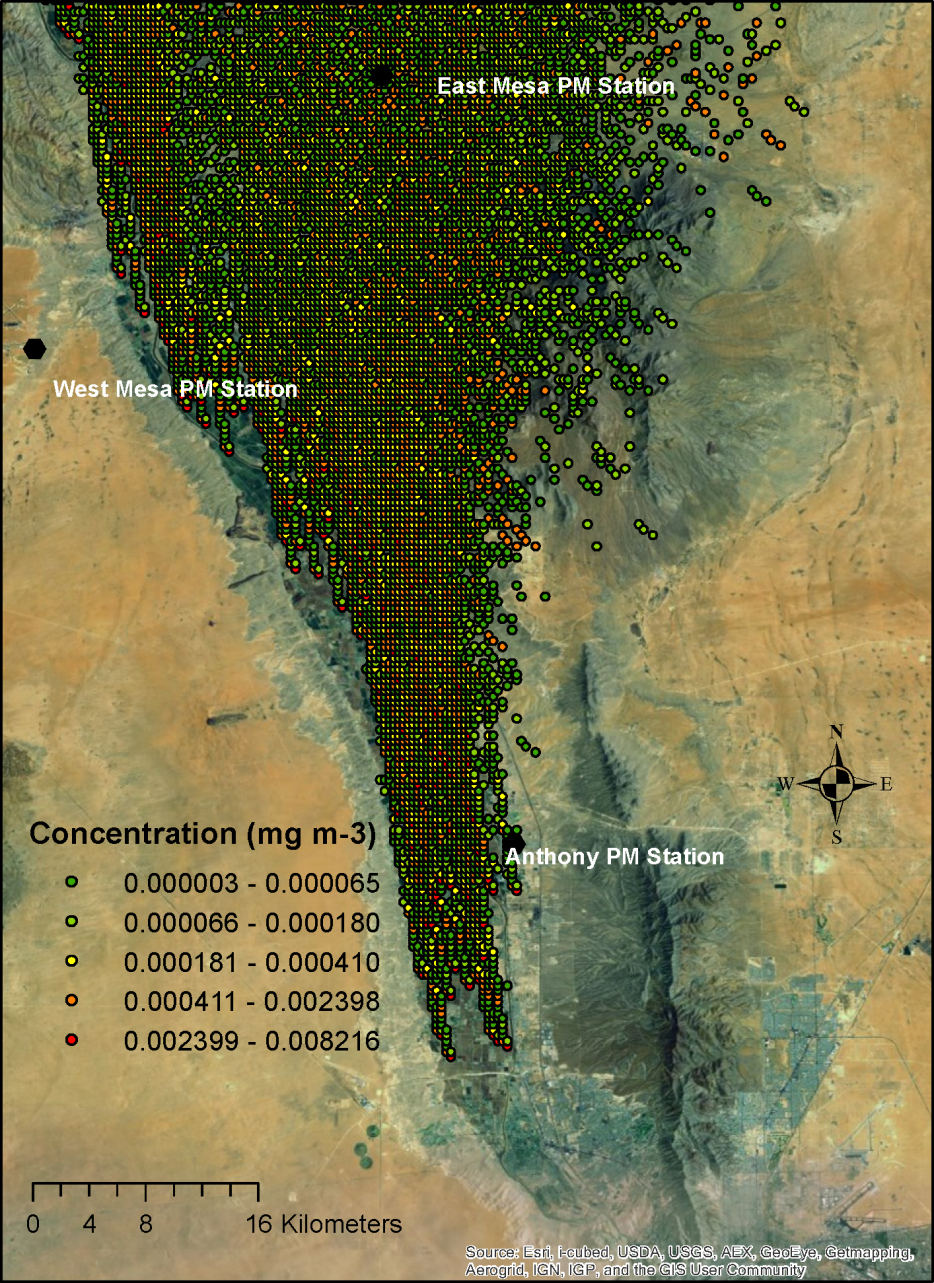
Superimposed PM10 plume at 13:00 MDT on April 1, 2008 from all potential tilling sources in the Mesilla Valley of southern New Mexico at a 2-m height (MDT: Mountain Daylight Time).

**Fig 5:**
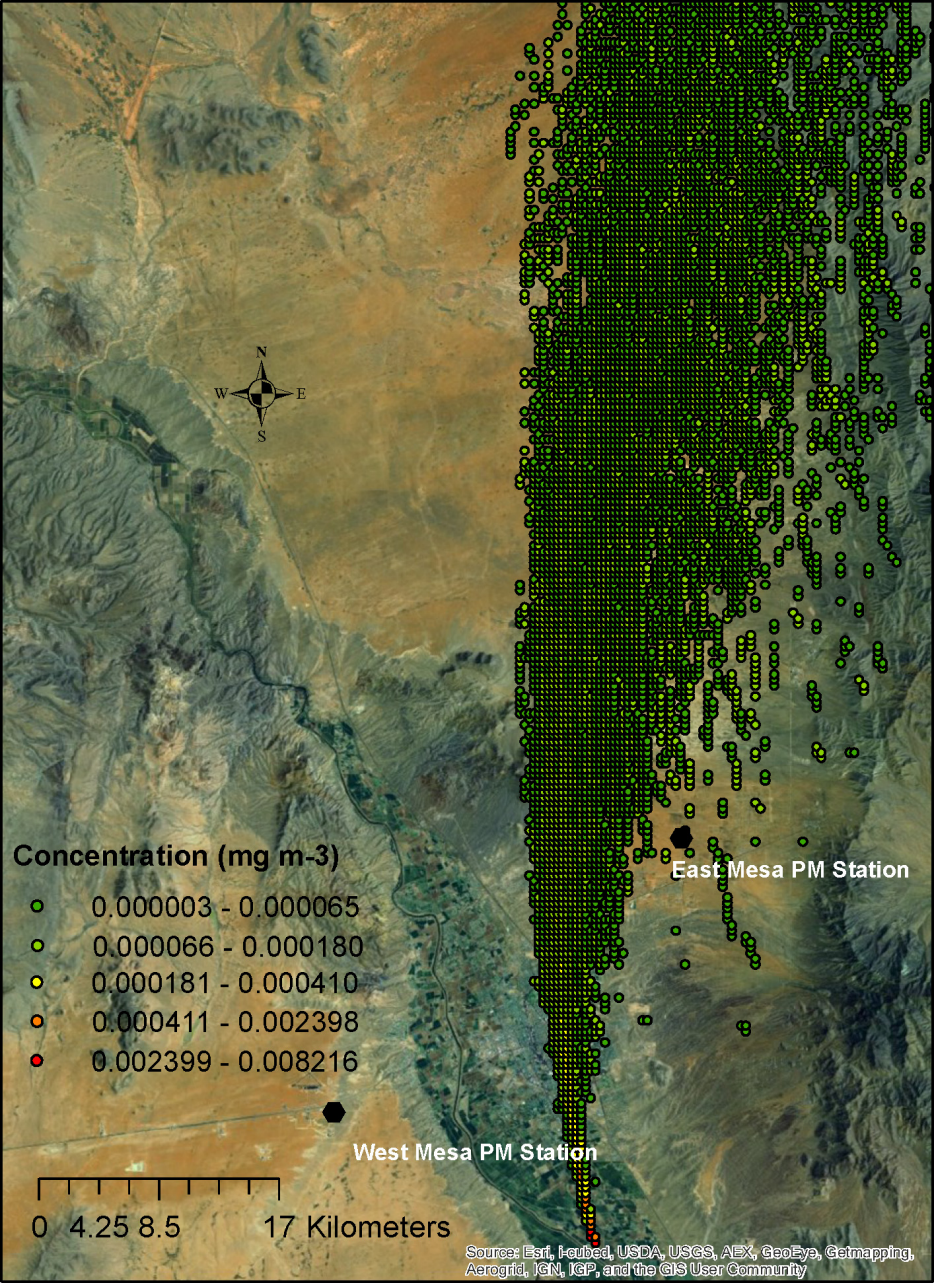
PM10 concentration plume at 200 m from a single tilling source at 13:13 MDT. on April 1, 2008 at the Leyendecker Research Farm in the Mesilla Valley of southern New Mexico (MDT: Mountain Daylight Time).

**Fig 6:**
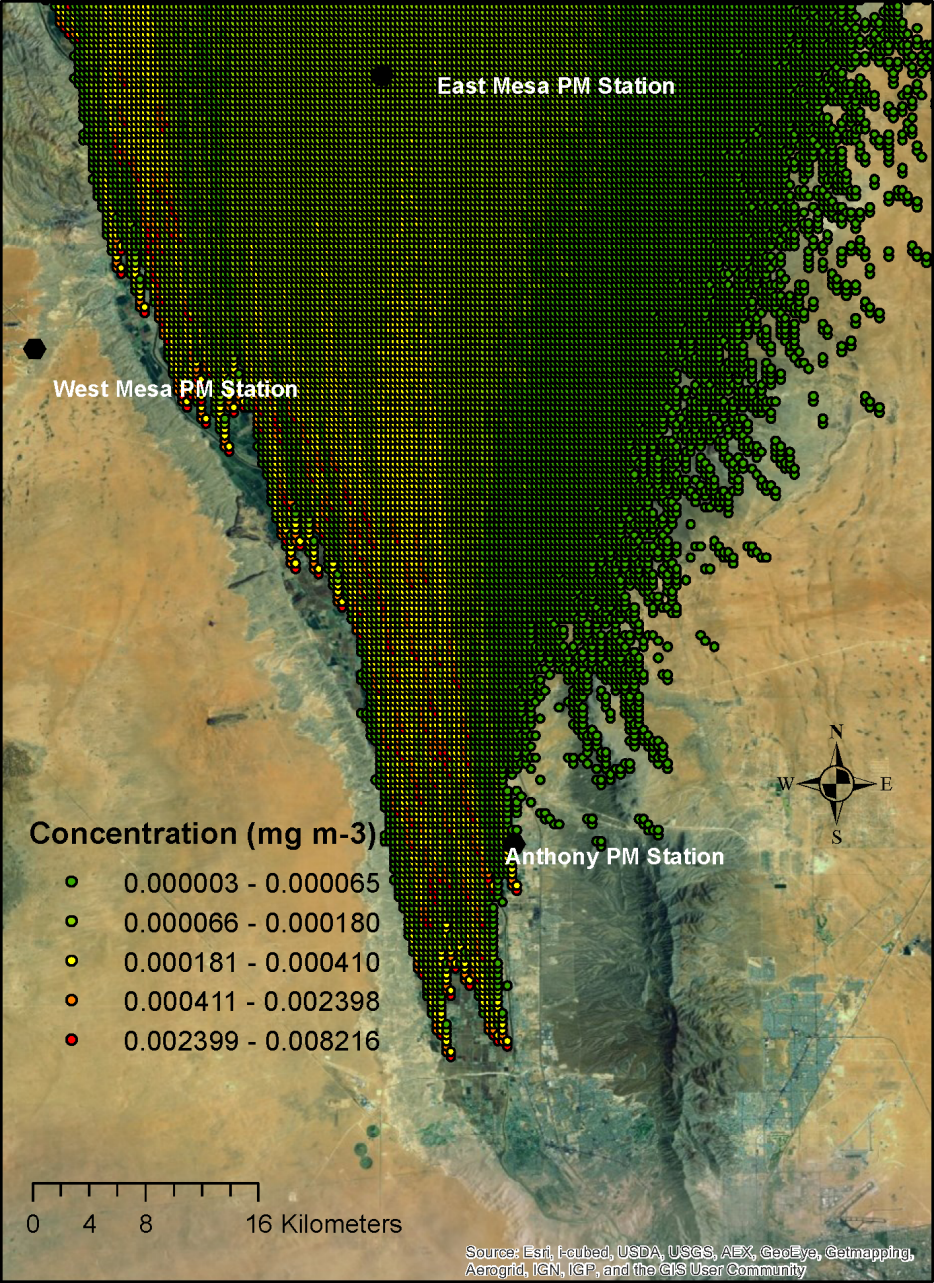
Superimposed PM10 plume at 200 m from all potential tilling sources at 13:13 MDT on April 1, 2008 in the Mesilla Valley of southern New Mexico (MDT: Mountain Daylight Time).

Fig 3 shows a sample figure of the simulated 2-m height dust concentration from a single source, and Fig 4 shows the concentrations with a superimposition of all potential sources. The gridded superimposed data closest to the air quality stations were compared with the corresponding measured PM10 data (superimposed data divided by the measured data) to calculate the percentage of contributions from agricultural operations for each hour for all three 2-m air quality sampler locations. As can be observed in Fig 3, a plume originates from a single source, and the location of the plume is determined by wind direction and intensity. When all sources of agricultural dust are combined (Figs 3–6), the concentration is higher in the valley as one moves from the edge of the valley to the center where more farming operations contribute to the dust concentration.

### Model verification

**Fig 7:**
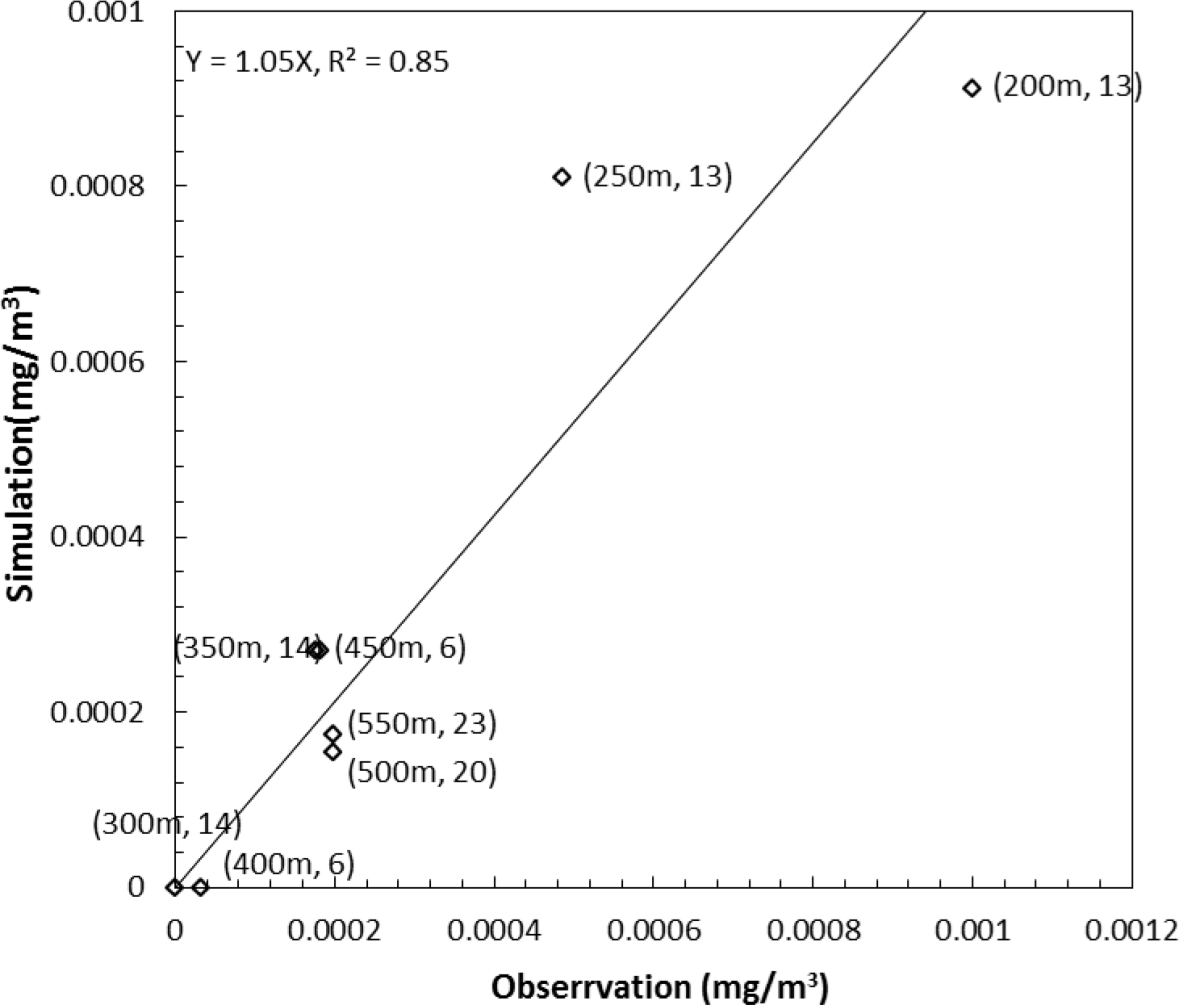
PM10 concentration comparison between observation and simulation at different heights from 11:13 MDT to 13:13 MDT on April 1, 2008 (MDT: Mountain Daylight Time). The concentration of the simulation at each height was calculated for the downwind plume area that intersected with the concentration of observation. Heights were from 200 to 550 m. The lables on the graph are heights following number of compared data points (e.g., 350 m, 14: The number of compared data points (number of corresponding observed data points to a compared simulation data point), was 6 at 450 m).

Using the experimental data, the model was verified (Fig 7) (please see details in [8]). Fig 7 shows the model simulation compared with the observation at different heights. The number of compared data points (number of corresponding measured data points to a compared simulation data point) ranged from 6 to 23. Generally, measured and simulated concentrations decrease with height. The linear regression shows that the simulated = 1.05×measured with R^2^ 0.85. Therefore, the HYSPLIT 4.0 model is capable of simulating the regional PM10 dispersion from agricultural tilling operations, given the appropriate meteorological inputs and calibrated parameters as long as the source strength input (emission rate) is accurate.

### Contribution ratio at 2 m height

**Fig 8:**
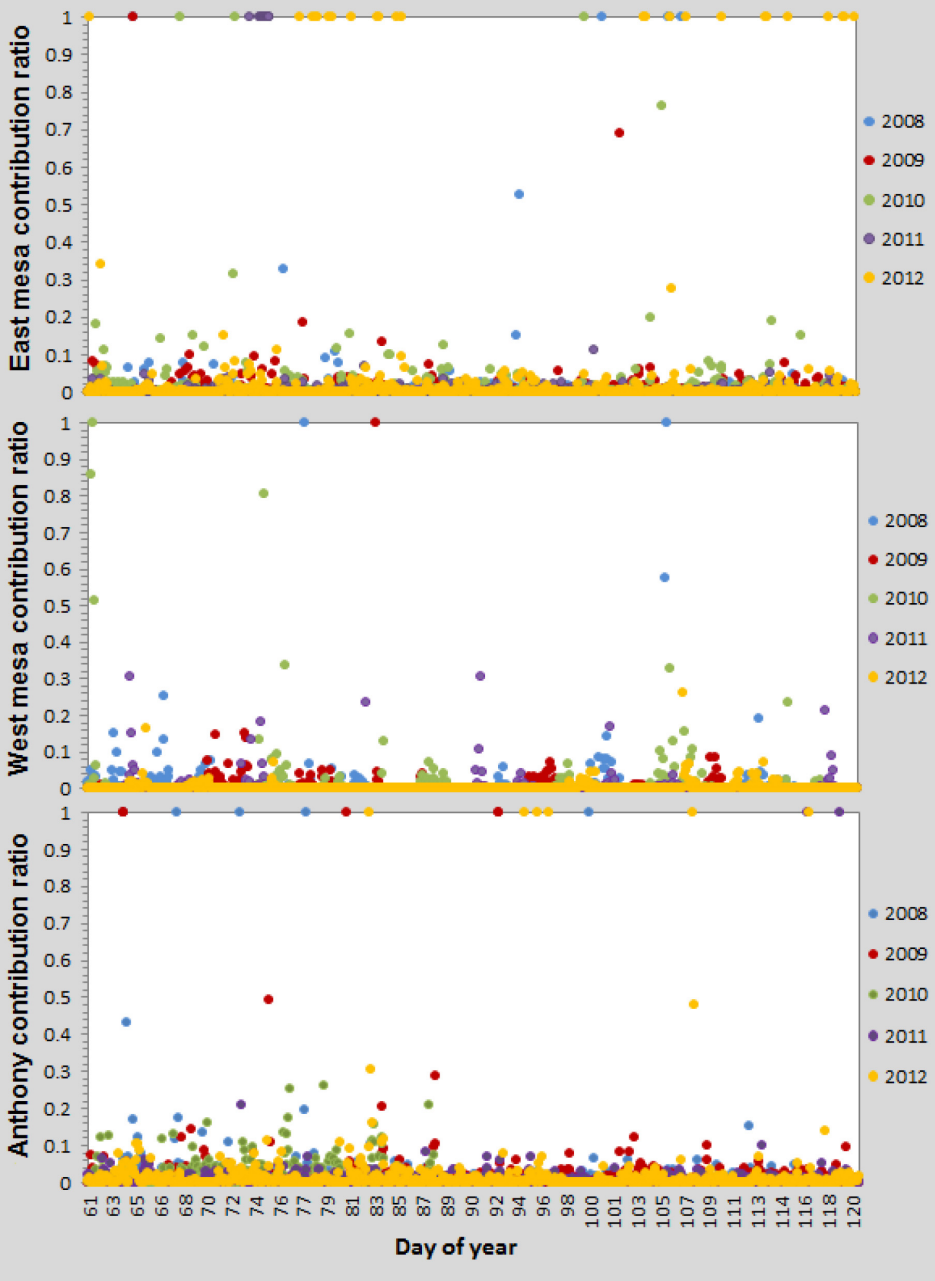
Hourly contribution ratio of modeled PM10 from agriculture to measured PM10 at 2 m for the years 2008–2012 for March through April. Top: at the east mesa site, middle: at the west mesa site, bottom: at the Anthony site.

**Fig 9:**
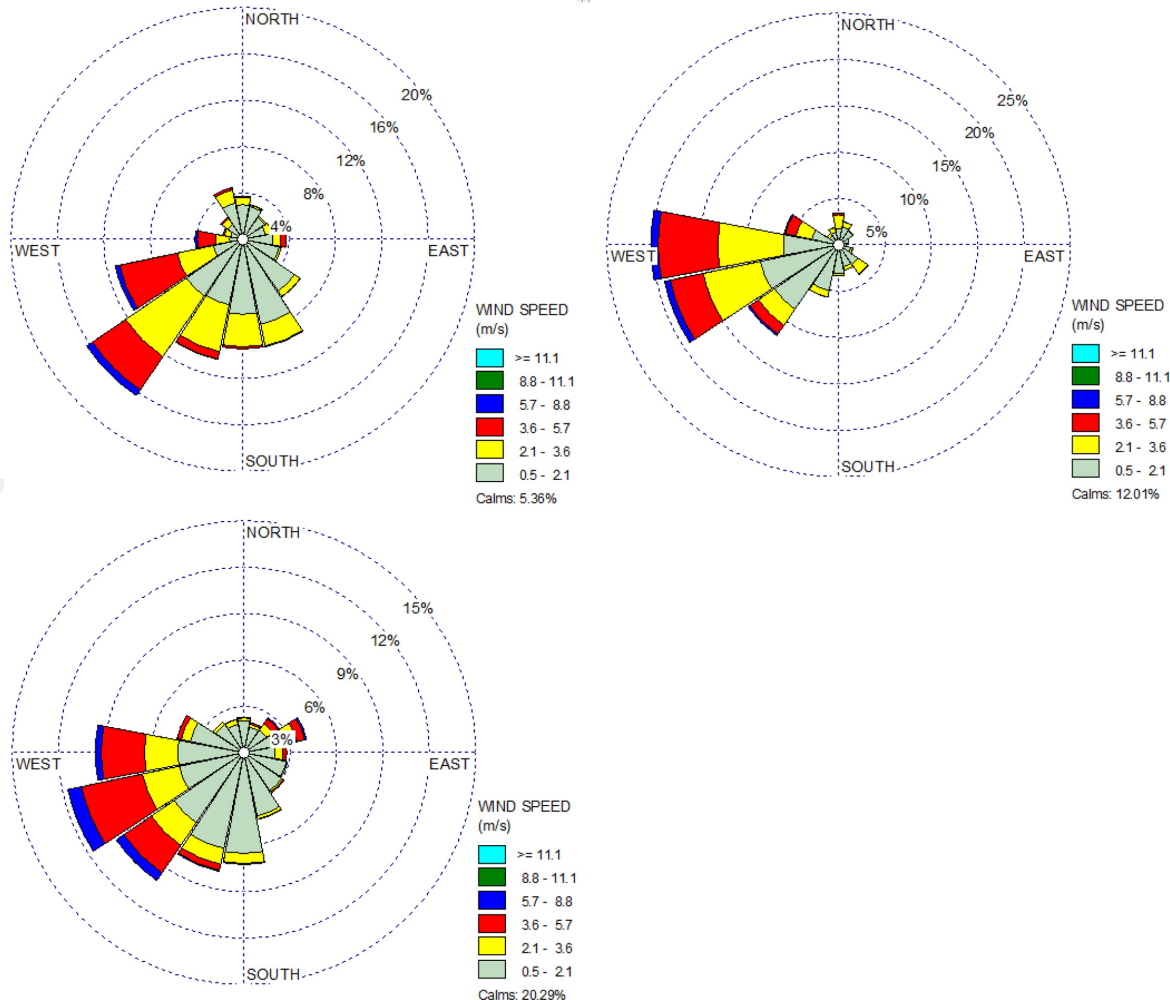
Wind rose 2008–2012 for March 1 to April 30. Wind direction is the direction from which it originates. The data were 1-hour data measured at the east mesa PM station. Top-left: east mesa, Top-right: west mesa, Bottom: Anthony.

Most of the new housing and business construction occurred in the east mesa. On average, that location had the highest dust contribution ratio among the three sites, ranging from 0 to 100% (39 hourly values >30% during the simulation period) (Table 1, Fig 8). However, the average contribution during the specified two months for five years was only 1.5%. During those months, most of the wind blew from the southwest to the northeast (Fig 9). The west mesa, in all years (Table 1), had a lower average dust contribution (0.7%) from agriculture compared to the east mesa because the prevailing wind blew in an easterly direction, and the valley dust was blown to the west mesa site much less frequently. The wind must blow from the northeast to the southwest for the dust from the valley to reach the west mesa site, which happened less frequently than for the east mesa (Fig 9). Consequently, the peak contribution days varied between the east mesa and west mesa sites (Fig 8), but the peak amounts were similar.

**Table 1:** Average ratio of modeled dust from agriculture to total dust for the west mesa, east mesa, and Anthony measurement sites for the months March through April during 2008–2012. Numbers in parentheses are the standard deviation.

Year	West Mesa	East Mesa	Anthony
2008	0.005 (0.024)	0.011 (0.083)	0.013 (0.075)
2009	0.005 (0.038)	0.008 (0.047)	0.015 (0.082)
2010	0.012 (0.080)	0.011 (0.065)	0.015 (0.053)
2011	0.006 (0.047)	0.017(0.012)	0.008 (0.048)
2012	0.009 (0.081)	0.027 (0.148)	0.015 (0.083)
Average	0.007 (0.055)	0.015 (0.099)	0.013 (0.071)

Homeowners living in the valley (Antony) had similar PM10 concentrations from agriculture when compared with homeowners on the east mesa, but more PM10 concentrations than homeowners on the west mesa (Table 1). Anthony is south (48 km) of the west mesa site near the southern end of the Mesilla Valley. Residents of the west mesa had less PM10 pollution from agricultural operations compared to those in the east mesa and in the Anthony sites.

### Contribution ratio at 200 m

The single-source PM10 concentrations for dust from the Leyendecker Research Farm from 11:13 to 13:13 MDT on April 1, 2008 (i.e., before the superimposition), for 200-m height, are plotted in Fig 5. The input Control and Setup.cfg files to run the HYSPLIT 4.0 are similar to Appendices 1 and 2, except that the running period was different. The PM10 at the 200-m height was dispersed downwind, with the highest concentrations, as expected, occurring downwind and nearest to the source. As it traveled downwind, the PM10 diffused and began to settle, resulting in decreased concentrations. The plume width increased with the downwind distance. Close to the source, the concentration was on the order of 0.001 mg/m^3^. The concentration farther downwind was on the order of 0.0001 to 0.000001 mg/m^3^. The grid field in the model was rectangular, and the surface was flat, which resulted in other agricultural sources at different locations having nearly identical dust plumes in the model.

The superimposed dust plume concentrations at 200 m from all potential tilling sources are plotted in Fig 6. On April 1, 2008, the aircraft flew at 200 m from 11:13 to 13:13 MDT. The maximum superimposed concentrations were on the order of 0.001 mg/m^3^. The average of the superimposed concentration values at the airplane sampling area was 0.0009 mg/m^3^ (standard deviation: 0.0008 mg/m^3^), while the DustTrak on the airplane sampled an average of 0.0048 mg/m3 (standard deviation: 0.0017 mg/m^3^) during 11:13 to 13:13 MDT. Thus, agricultural tilling contributed about 19% of the regional PM10 concentration (19% = 0.009/0.0048) on the day of the airplane measurements at 200 m. The other 81% of concentrations were from other sources, such as emissions from construction sites, unpaved and paved roads, windblown dust, factories, wood burning, and concentrated animal feeding operations.

The contribution ratio for the point sites and the airplane results provide information on the amount that agricultural operations contribute to the total PM10 concentrations in the Mesilla Valley and in the east and west mesa, but several assumptions and sources of variability must be considered to provide confidence in the results.

### Source of errors

The model simulation represents a dry soil condition of 0.0234 g H_2_O/ g. If rainfall is above average during the season, and the actual soil moisture content is higher than in the model, the actual source strength would be lower and the contribution of dust from agriculture would be lower than estimated in this paper.

Another assumption was that 120 tractors operated for 12 hours each day in the valley. There may have been more or fewer numbers of tractors owned and hours operated by the farmers. Assuming tractor operations are relatively independent events with respect to each other, it follows from Poisson statistics that the observed number of tractors operating in the Mesilla Valley should deviate from the average depending on the square root of the average number of tractors in operation. Using the number of tractors modeled (120) as approximately the average number of tractors in operation in the Mesilla Valley, the observed variability in the number of tractors in operation should be on the order of ~10%. A conservative estimate is that a ~10% variability in dust concentrations is to be expected. Additionally, the diurnal variability can be on the order of ~30% [17]. This is due to the variability of human activity from day to night.

Local spatial variability also needs to be considered. This is because the variability will be detectable as an instrument was moved through the air taking dust measurements, or as the wind carried the spatially variable dust concentrations past a stationary instrument. The standard deviation in the modeled concentration over the airplane sampling area at 2 m and 200 m provides an estimate of ~20%, which can be assumed to represent the spatial variability. Assuming all sources of variability mentioned are uncorrelated, the variability should approximately add in quadrature, resulting in a total variability of ~40%.

When the HYSPLIT 4.0 was run for each day between March 1 and April 30, 2008–2012, and was compared with the measured data, the percentage of concentrations contributed by agricultural dust was highly variable (coefficient of variation was 7.9, 6.6, and 5.4 for the west mesa, east mesa, and Anthony sites, respectively) depending on the wind direction and intensity. Consequently, daily variability in wind speed and wind direction had a large effect on the magnitude of the contribution of agriculture operations on the quality of the air in the new subdivisions on the east and west mesas and in the valley at Anthony.

The contribution of agricultural dust at Anthony at 2 m at 11:00 to 13:00 MDT on the same day as the airplane measurements was also 19%, supporting the reliability of the simulated data compared with measured data. If the assumptions stated in the paper on the number of tractors and their location and the source strength of the dust from the tractors were erroneous, the simulated and measured data from the airplane measurements would not have been similar. Based on the simulations, it appears that agriculture on certain days does contribute substantially to the overall dust problem.

On windy days with the wind in a certain direction, the contribution can be as high as 30 to 100%. Before regulating farming operations for dust contribution, additional studies need to be conducted on these high-dust days to evaluate the amount of the total PM10 that is contributed from unpaved roads and construction sites, which currently are regulated.

Agricultural operations also generate dust during harvest, and a separate study would need to be conducted in the fall to determine the impact of harvest operations on dust concentrations. However, during that time the wind velocities typically are low, and most of the dust would likely stay in the valley, causing problems for valley residents but not for the east mesa and west mesa residents.

## Concluding remarks

This paper developed a method that combines measurements and modeling to estimate the proportion of field agricultures’ contribution to the total PM10 from all regional sources. Depending on wind speed, direction, and location, the agricultural operations’ contribution to total airborne PM10 at a 2-m height were shown to be as high as 30 to 100% on extreme days. On average, for the two agricultural operation months (March and April) during 2008 to 2012, the contribution was as low as 0.7%, 1.5%, and 1.3%,for the upwind (west mesa), downwind (east mesa), and source area (valley center at Anthony) sites, respectively. Agricultural operations significantly affected regional air quality on individual days, yet depending on wind speed and direction during the season, the agriculture field operation contributions were relatively low.

Additional research should quantify the weather conditions that result in high proportional PM10 contributions from agriculture before agricultural farming operations that create dust become regulated.

## Appendix 1

The sample Control file was used for the model implementation. For a detailed explanation of the parameters definition, check the HYSPLIT user guide (http://www.arl.noaa.gov/documents/reports/hysplit_user_guide.pdf).

08 04 11 15 Comments: the date and year and Coordinated Universal Time to start the simulation.

1 Comments: number of start locations.

32.197147–106.7385139 5 Comments: start location and release height.

240 Comments: total run time (hours).

0 Comments: Use the NAM12KM vertical velocity fields.

10000.0 Comments: top model domain (m) above ground level.

10 Comments: number of meteorological files used in calculation.

N:/NAM/2008/4/20080411_nam12 Comments: directory location of the meteorological file.

N:/NAM/2008/4/20080412_nam12

N:/NAM/2008/4/20080413_nam12

N:/NAM/2008/4/20080414_nam12

N:/NAM/2008/4/20080415_nam12

N:/NAM/2008/4/20080416_nam12

N:/NAM/2008/4/20080417_nam12

N:/NAM/2008/4/20080418_nam12

N:/NAM/2008/4/20080419_nam12

N:/NAM/2008/4/20080420_nam12

7 Comments: seven classes of particle sizes.

C01_ Comments: Class 1 particle configuration.

0.01082833 Comments: emission rate (one/hr), unitless here. The simulation results should be scaled by the true source release rate.

12 Comments: hours of emission.

08 04 11 15 00 Comments: start release time of particles.

C02_ Comments: Class 2 particle configuration

0.004668807.

12

08 04 11 15 00

C03_ Comments: Class 3 particle configuration

0.01459364.

12

08 04 11 15 00

C04_ Comments: Class 4 particle configuration

0.153797846.

12

08 04 11 15 00

C05_ Comments: Class 5 particle configuration

0.634348714.

12

08 04 11 15 00

C06_ Comments: Class 6 particle configuration

0.051306124

12

08 04 11 15 00

C07_ Comments: Class 7 particle configuration

12

08 04 11 15 00

1 Comments: number of grid size.

0.0 0.0 Comments: center is at the release location.

0.003 0.003 Comments: the spacing (degree).

3 3 Comments: span (degree)

./ Comments: Output directory.

Test data Comments: output grid file name.

13 Comments: number of vertical layers.

0 2 150 200 250 300 350 400 450 500 550 600 650 Comments: height of levels (m)

00 00 00 00 00 Comments: sampling start year, month, day, hour, minute, (the setting means to start from the simulation start time).

00 00 00 00 00 Comments: sampling stop; year, month, day, hour, minute (the setting means to stop at the end of the simulation).

00 01 00 Comments: output the average data every hour.

7 Comments: seven classes of particles for deposition setup.

5.0 6.0 1.0 Comments: particle physical size for Class 1 particle. (It is not valid if the following settling speed is set.)

0.006 0.0 0.0 0.0 0.0 Comments: settling speed is 0.006 m/s.

0.0 0.0 0.0 Comments: Henry’s (M/a), In-cloud (l/l), Below-cloud (l/s).

10960.0 Comments: radioactive decay half-life (days).

0.0 Comments: pollutant resuspension factor (1/m).

0.621 2.0 1.0 Comments: particle physical size for Class 2 particle.

0.000015 0.0 0.0 0.0 0.0

0.0 0.0 0.0

10960.0

0.0

0.7721 2.0 1.0 Comments: particle physical size for Class 3 particle.

0.000038 0.0 0.0 0.0 0.0

0.0 0.0 0.0

10960.0

0.0

1.2471 2.0 1.0 Comments: particle physical size for Class 4 particle.

0.000098 0.0 0.0 0.0 0.0

0.0 0.0 0.0

10960.0

0.0

2.0 2.0 1.0 Comments: particle physical size for Class 5 particle

0.00034 0.0 0.0 0.0 0.0

0.0 0.0 0.0

10960.0

0.0

4.0146 2.0 1.0 Comments: particle physical size for Class 6 particle

0.001 0.0 0.0 0.0 0.0

0.0 0.0 0.0

10960.0

0.0

8.1853 2.0 1.0 Comments: particle physical size for Class 7 particle

0.0042 0.0 0.0 0.0 0.0

0.0 0.0 0.0

10960.0

0.0

## Appendix 2

The sample Setup.cfg file used for the model implementation. For a detailed explanation of the parameters definition, check the HYSPLIT user guide (http://www.arl.noaa.gov/documents/reports/HYSPLIT_user_guide.pdf).

&SETUP

tratio = 0.75, Comments: defines 75% of a grid cell that a particle is permitted to transit in in advection time step.

initd = 0, Comments: 3D particle horizontal and vertical model.

kpuff = 0, Comments: Particle model will be converted to horizontal puff and vertical puff model after 48 hours simulation (not applicable here).

khmax = 9999, Comments: The maximum particle duration is the number of hours after release a particle is dropped from the simulation.

kmixd = 0, Comments: use HYSPLIT model to calculate boundary layer depth.

kmix0 = 250, Comments: minimum mixing depth is set to 250 m.

kzmix = 0, Comments: None, vertical diffusivity in planetary boundary layer varies with height.

kdef = 0, Comments: Horizontal turbulence is in proportion to the vertical turbulence.

kbls = 1, Comments: Stability calculation is based on heat and momentum fluxes.

kblt = 2, Comments: vertical turbulence computational method: Kanthar/Clayson.

conage = 48, Comments: hours for particle conversion into the global model.

numpar = 25000, Comments: the number of particles released per cycle.

qcycle = 24, Comments: the number of hours between emission start cycles.

efile = '', Comments: the file name that contains point-source temporal emission factors (undefined).

tkerd = 0.18, Comments: the ratios of the vertical to the horizontal turbulence for daytime.

tkern = 0.18, Comments: the ratios of the vertical to the horizontal turbulence for nighttime.

ninit = 1, Comments: reads the “PINPF” file only during the initialization.

process; *PINPF*sets the default name *PARINIT* for the particle input file.

ndump = 0, Comments: for no I/O or [hours] to set the number of hours.

from the start of the simulation at which all the endpoint positions will be written to the file. ncycl = 0. Comments: sets the repeat interval at which the *PARDUMP* file is to be written after the first write at hours.

pinpf = 'PARINIT', Comments: default name for the particle input file.

poutf = 'PARDUMP', Comments: default name for the particle dump output file.

mgmin = 10, Comments: the minimum size in grid units of the meteorological sub-grid.

kmsl = 0, Comments: input heights to be relative to the terrain height of the meteorological model.

maxpar = 100000, Comments: the maximum number permitted to be carried at any time during a simulation.

cpack = 1, Comments: to write the binary concentration file at only those grid points that have a non-zero concentration value.

cmass = 0, Comments: to output units of mass/volume.

dxf = 1.0, Comments: the west-to-east grid factor for offsetting the meteorological grid in the ensemble calculation.

dyf = 1.0, Comments: the south-to-north grid factor for offsetting the meteorological grid in the ensemble calculation.

dzf = 0.01, Comments: the vertical grid offset factor (m).

ichem = 0, Comments: no chemistry module selection.

kspl = 1, Comments: the interval in hours at which the puff splitting routines are called.

krnd = 6, Comments: the interval in hours at which enhanced puff merging takes place.

frhs = 1.0, Comments: the horizontal distance between puffs in sigma units that puffs have to be within each other to be permitted to merge.

frvs = 0.01, Comments: the vertical distance between puffs in sigma units that puffs have to be within each other to be permitted to merge.

frts = 0.1, Comments: the fractional difference in the age that puffs have to be within each other to be permitted to merge.

frhmax = 3.0, Comments: the maximum permissible value for FRHS.

splitf = 1.0, Comments: at the default value of 1.0 is automatically recomputed to be the ratio of the number of concentration grid cells to the maximum number of particles permitted.

/, Comments: the Setup.cfg file end symbol.
